# Online co-care management and glycemic control in patients with type 2 diabetes mellitus: A propensity score-matched retrospective cohort study

**DOI:** 10.1371/journal.pone.0360677

**Published:** 2026-11-02

**Authors:** Shujuan Sun, Ge Yang, Hao Tong, Hui Zhao, Qin Liu, Yiran He, Zhiyue Zhang

**Affiliations:** 1 General Practice Department, Wanshou Road Community Health Service Center of Haidian District, Beijing, China; 2 Department of Clinical Nutrition, Union Hospital, Tongji Medical College, Huazhong University of Science and Technology, Wuhan, China; 3 Department of Traditional Chinese Medicine, Air Force Medical Center PLA, Beijing, China; 4 General Practice Department, Wanshou Road Community Health Service Center of Haidian District, Beijing, China; 5 General Practice Department, Air Force Medical Center PLA, Beijing, China; Charite Universitatsmedizin Berlin, GERMANY

## Abstract

**Objective:**

This study aimed to investigate the association of an Online Co-care Management (OCCM) model with changes in glycemic control and metabolic indicators among patients with type 2 diabetes mellitus (T2DM).

**Methods:**

Based on clinical follow-up records, we conducted a retrospective cohort study with a propensity score (PS)-matched control group.The OCCM group included patients enrolled in the integrated care clinic from 2022 to 2025 (n = 406). Among patients who did not participate in OCCM (n = 199) during the study period, PS-matched controls were selected at a 1:1 ratio.The PS model included age, sex, diabetes duration, smoking, drinking, medication use, and follow-up duration.After matching, 240 patients were included in the final analysis, with 120 patients in each group. Follow-up data were collected at baseline, 3 months, and 6 months. Longitudinal data on anthropometrics, glycemic indices, blood pressure, lipid profiles, and organ function were analyzedusing repeated measures ANOVA.

**Results:**

Over a 6-month follow-up period, HbA1c levels decreased more markedly in the OCCM group compared with the usual-care group. In the OCCM group, HbA1c declined from 7.403 ± 1.748% at baseline to 6.591 ± 0.871% at 3 months and 6.593 ± 1.056% at 6 months, while in the usual-care group, it changed from 6.310 ± 1.277% to 6.225 ± 1.017% and 6.534 ± 1.598%, respectivelyRepeated-measures ANOVA revealed significant time, group, and time × group effects for HbA1c (F = 10.608, P < 0.001; F = 16.748, P < 0.001; F = 14.546, P < 0.001). BMI, systolic and diastolic blood pressure, and lipid profiles, also showed greater improvements in the OCCM group than in controls.

**Conclusion:**

The OCCM model was associated with short-term improvements in glycemic and metabolic indicators among T2DM patients, suggesting it may be an effective approach for structured chronic disease management.

## Introduction

Type 2 diabetes mellitus (T2DM) is a chronic metabolic disorder characterized by insulin resistance and β-cell impairment [1,2]. Driven by rapid urbanization and population aging, the number of adults with diabetes in China reached 114 million in 2017 andhas continued to increase [3,4]. Effective management of this condition necessitates not only glycemic controlbut also attention to cardiovascular risk factors, including hypertension and dyslipidemia, which are closely related to diabetes-related complications [5]. However, traditional outpatient care models are oftenlimited by fragmented services and intermittent visits, limiting the ability of healthcare providers to offer timely and continuous support for patient self-management.

To bridge the gap between clinical visits and daily self-care, mobile health (mHealth) technologies have been increasingly adopted. Previous studies [6,7] have suggested that various e-health approaches are associated with short-term improvements in HbA1c among patients with T2DM. However, technology alone may not be sufficient to support sustained self-management or address complex clinical needs [8].Diabetes care often requires coordination among different healthcare professionals, together with active patient participation. In this context, patient-centered collaborative care, shared decision-making, and the Online Co-care Management (OCCM) model featuring deep patient engagement are considered more conducive to addressing complex diabetes management issues [9,10]. Carolina et al. [11] explored how chronic disease patients experience typical configurations of co-care systems that support self-monitoring and digital communication with primary care based on the co-care model. Wei Wulin et al. [12]reported that an online-offline co-care model was associated with better glycemic management in pregnant women with hyperglycemia. Furthermore, Su et al. [13]also found that higher levels of patient activation and engagement with remote monitoring technologies were correlated with better glycemic control.Although such collaborative models have demonstrated advantages in glycemic management, existing literature predominantly focuses on short-term glycemic outcomes,while comparative evidence on metabolic indicators in routine clinical practice remains limited.

To address these research gaps, the present study used a retrospective cohort design with a propensity score-matched usual-care control group to evaluate an Online Co-care Management (OCCM) model among patients with T2DM in a real-world clinical setting. By analyzing follow-up data at baseline, 3 months, and 6 months, this study aimed to examine whether changes in HbA1c and secondary metabolic indicators differed between patients receiving OCCM and those receiving usual outpatient follow-up care. The findings may provide practical evidence for the use of collaborative digital care models in diabetes management.

## Method

### Subjects

We conducted a retrospective cohort study with a propensity score-matched control group from September 2022 to September 2025. The study included patients with type 2 diabetes mellitus (T2DM) who received Online Co-care Management (OCCM) and those who received usual outpatient follow-up care. Inclusion criteria were: (1) diagnosis of T2DM according to Guideline for the prevention and treatment of type 2 diabetes mellitus in China (2020 edition) [14]; (2) diabetes duration ≥6 months; and (3) availability of follow-up data during the study period. Exclusion criteria were: (1) age < 18 years; (2) acute diabetic complications, such as diabetic ketoacidosis, hyperosmolar hyperglycemic state, or severe hypoglycemia; (3) other endocrine disorders, such as hyperthyroidism or hypercortisolism; (4) history of respiratory diseases, including chronic bronchitis or chronic obstructive pulmonary disease; (5) history of acute or chronic pancreatitis or pancreatic cancer; (6) hematologic conditions, including trauma, acute infection, or anemia, with alanine aminotransferase (ALT) >120 U/L and/or aspartate aminotransferase (AST) >120 U/L; (7) estimated glomerular filtration rate (eGFR) <45 mL·min^−1^·1.73 m^−2^; and (8) other personal reasons preventing participation.

Patients in the OCCM and usual-care groups were matched 1:1 using propensity scores. The propensity score model included age, sex, diabetes duration, smoking status, drinking status, medication use, and follow-up duration. After matching, 240 patients were included in the final analysis, with 120 in each group. Follow-up data were collected at three time points: baseline, 3 months, and 6 months, defined as T0, T1, and T2, respectively. The primary outcome was glycated hemoglobin (HbA1c). Secondary outcomes included body mass index (BMI), blood pressure, lipid profiles, and renal function indicators. This retrospective study was based on routinely collected clinical data and was approved by the hospital ethics committee (Approval No. ZXYEC-KT-2024-07-P01).

### Online co-care management model

The Online Co-care Management (OCCM) model establishes an integrated, one-stop service framework involving the patient, one physician, two diabetes specialist nurses, an ophthalmologist for retinal screening, and assistant nurses. The responsibilities of each team member are clearly defined and documented. All team members receive standardized training on relevant clinical knowledge and the use of the system software to ensure accurate implementation. A WeChat group is created for collaborative communication, facilitating timely interaction and information exchange among team members.

All team members receive standardized training on relevant knowledge and the use of system software to ensure correct implementation. A WeChat group for collaborative care communication is established to facilitate interaction and information exchange among members. After establishing a health record in the outpatient clinic, patients attend appointments on a scheduled basis. Each patient enrolled in the integrated care clinic participates in quarterly diabetes-related health education sessions. Laboratory tests for HbA1c, blood glucose, and renal function (eGFR and UA) are performed according to the follow-up schedule. Diet, physical activity, medication adherence, and blood glucose monitoring are assessed selectively based on follow-up results, and tailored educational guidance is provided.

Each member of the collaborative care team performs distinct responsibilities: (i) Physicians adjust treatment plans based on the patient’s same-day test results and monitor trends in laboratory findings compared to previous visits, providing corresponding notifications and guidance to patients. (ii) Diabetes specialist nurses use the education management documentation module, following the framework of subjective description, objective description, diagnosis, and plan, to assess patients’ medication use and lifestyle habits, and deliver targeted intervention and guidance. (iii) Assistant nurses are responsible for measuring vital signs and conducting physical examinations.

All examination results, medical records, and educational materials are electronically stored in the collaborative care system, enabling other team members to access and share patient information promptly, thereby supporting comprehensive and systematic patient management. Throughout the process, team members provide information, education, and support while respecting patients’ autonomy, without imposing decisions.

### Data collection and evaluation indicators

A detailed medical history and physical examination were conducted for each patient, and the following data were recorded: (i) Demographic characteristics: sex, age, smoking (yes/no), drinking (yes/no), hypertension, and hyperlipidemia. (ii) Medication history: insulin use (yes/no) and oral antidiabetic medications including biguanides, sulfonylureas, α‑glucosidase inhibitors, and DPP‑4 inhibitors. (iii) Primary outcome: glycated hemoglobin (HbA1c). (iv) Secondary outcomes: body mass index (BMI), systolic blood pressure (SBP), diastolic blood pressure (DBP), lipid profiles, and renal function indicators. Lipid profiles included triglycerides (TG), total cholesterol (TC), low-density lipoprotein cholesterol (LDL-C), and high-density lipoprotein cholesterol (HDL-C). Renal function indicators included estimated glomerular filtration rate (eGFR) and uric acid (UA).All data were collected from routinely recorded clinical follow-up records. After approval from the hospital administration, the relevant data were downloaded, checked, and prepared for statistical analysis.

### Statistical analysis

Propensity scores were estimated using a logistic regression model including age, sex, diabetes duration, smoking (yes/no), drinking (yes/no), medication use, and follow-up duration as covariates. Each patient in the OCCM group was matched 1:1 with a patient in the usual-care group using a nearest-neighbor algorithm without replacement, with a caliper width of 0.2. For repeated outcome measurements, repeated-measures ANOVA was performed. When the assumption of sphericity was violated, Roy’s largest root test was applied, followed by post hoc multiple comparisons. Continuous variables were expressed as mean ± standard deviation (Mean ± SD), and categorical variables were expressed as percentages, as appropriate. A two-sided P value less than 0.05 was considered statistically significant. All analyses were performed using SPSS version 26.0.

## Results

### Baseline characteristics

Table 1 presents the baseline characteristics of the OCCM and usual-care groups. All follow-up records of patients with type 2 diabetes from 2022 to 2025 were extracted (n = 977). After excluding patients with dropout, loss to follow-up, extreme values, or records that met the exclusion criteria, 605 patients were included in the original cohort, including 406 patients in the OCCM group and 199 patients in the usual-care group. After propensity score matching, 240 patients were included in the final analysis, with 120 patients in each group. The participant selection process is shown in S1 File.

**Table 1:** Clinical and demographic characteristics (n = 240).

Variables		Total (n = 240)	Usual-care group (n = 120)	OCCM group (n = 120)	*t/X* ^*2*^	*P*
Age		68.32 ± 8.517	68.43 ± 8.807	68.21 ± 8.253	0.042	0.838
Disease duration (years)		11.981 ± 8.601	11.758 ± 8.772	12.204 ± 8.458	0.161	0.689
Gender	Male	133(55.417)	68(56.667)	65(54.167)	0.152	0.697
	Female	107(44.583)	52(43.333)	55(45.833)		
Drinking	No	173(72.083)	85(70.833)	88(73.333)	1.294	0.523
	Yes	66(27.500)	35(29.167)	31(25.833)		
Smoking	No	190(79.167)	93(77.500)	97(80.833)	0.404	0.525
	Yes	50(20.833)	27(22.500)	23(19.167)		
Hypertension	No	65(27.083)	30(25.000)	35(29.167)	0.527	0.468
	Yes	175(72.917)	90(75.000)	85(70.833)		
Hyperlipidemia	No	58(24.167)	29(24.167)	29(24.167)	0	1
	Yes	182(75.833)	91(75.833)	91(75.833)		
Biguanides	No	94(39.167)	47(39.167)	47(39.167)	0	1
	Yes	146(60.833)	73(60.833)	73(60.833)		
Sulfonylureas	No	212(88.333)	108(90.000)	104(86.667)	0.647	0.421
	Yes	28(11.667)	12(10.000)	16(13.333)		
AGIs	No	155(64.583)	78(65.000)	77(64.2)	0.018	0.893
	Yes	85(35.417)	42(35.000)	43(35.833)		
DPP-4 inhibitors	No	156(65.000)	79(65.833)	77(64.167)	0.073	0.787
	Yes	84(35.000)	41(34.167)	43(35.833)		
insulin	No	229(95.417)	114(95.000)	115(95.833)	0.095	0.758
	Yes	11(4.583)	6(5.000)	5(4.167)		

As shown in Table 1, the mean age of the matched cohort was 68.32 ± 8.52 years, and the mean duration of diabetes was 11.98 ± 8.60 years. The two groups were well balanced in sex distribution, with 56.7% male patients in the usual-care group and 54.2% in the OCCM group (*P* = 0.697). No significant differences were observed between the usual-care and OCCM groups in smoking status (22.5% and 19.2%, respectively; *P* = 0.525), drinking status (29.2% and 25.8%, respectively; *P* = 0.523), hypertension (75.0% and 70.8%, respectively; *P* = 0.468), or hyperlipidemia (75.8% in both groups; *P* = 1.000). The use of antidiabetic medications, including biguanides, sulfonylureas, α-glucosidase inhibitors, DPP-4 inhibitors, and insulin, was also comparable between groups. Overall, no statistically significant differences were found in the baseline characteristics, indicating good balance after matching.

### Repeated measures ANOVA results

#### Primary outcome.

After three months of online co-care management, HbA1c levels in the OCCM group decreased from 7.403 ± 1.748% at baseline (T0) to 6.591 ± 0.871% at 3 months (T1) and 6.593 ± 1.056% at 6 months (T2). In the control group, HbA1c decreased from 6.310 ± 1.277% at baseline to 6.225 ± 1.017% at 3 months and 6.534 ± 1.598% at 6 months (Table 2).

**Table 2:** Characteristics of Primary and Secondary Indicators of Glycemic Management in the Two Groups.

Variables	Baseline		Follow-up 3 months		Follow-up 6 months	
	OCCM group	Usual-care group	OCCM group	Usual-care group	OCCM group	Usual-care group
HbA1c	7.403 ± 1.748	6.310 ± 1.277	6.591 ± 0.871	6.225 ± 1.017	6.593 ± 1.056	6.534 ± 1.598
SBP	132.833 ± 13.480	126.017 ± 6.657	127.800 ± 11.337	126.417 ± 6.225	126.858 ± 9.350	125.758 ± 5.773
DBP	77.308 ± 8.214	74.225 ± 3.873	75.017 ± 6.679	74.933 ± 4.443	74.717 ± 5.534	74.825 ± 4.123
BMI	25.165 ± 3.280	25.073 ± 1.701	24.893 ± 3.238	25.028 ± 1.811	24.707 ± 3.214	25.195 ± 1.635
LDLC	2.322 ± 3.181	2.834 ± 1.148	2.320 ± 0.822	2.724 ± 0.740	2.203 ± 0.847	2.775 ± 0.781
HDLC	1.264 ± 0.328	1.339 ± 0.395	1.265 ± 0.352	1.301 ± 0.303	1.320 ± 0.344	1.310 ± 0.287
TC	4.579 ± 1.145	5.028 ± 1.398	4.199 ± 1.210	4.801 ± 1.181	4.090 ± 1.322	4.723 ± 1.055
TG	1.714 ± 1.629	1.694 ± 1.099	1.559 ± 1.030	1.765 ± 1.503	1.348 ± 0.935	1.475 ± 0.987
eGFR	88.999 ± 14.759	89.327 ± 20.118	88.408 ± 15.535	88.920 ± 18.185	89.831 ± 18.189	92.765 ± 16.214
UA	315.115 ± 85.299	329.992 ± 92.281	324.626 ± 89.741	346.100 ± 89.741	303.298 ± 113.925	334.550 ± 90.000

Abbreviations used: HbA1c, hemoglobin A1c; BMI, Body Mass Index; SBP, Systolic Blood Pressure; DBP, Diastolic Blood Pressure; LDLC, Low-Density Lipoprotein Cholesterol; HDLC, High-Density Lipoprotein Cholesterol; TC, Total Cholesterol; TG, Triglycerides; eGFR, estimated glomerular filtration rate; UA, uric acid; Data are presented as mean ± standard deviation.

The changes in HbA1c levels over time in the OCCM and usual-care groups are shown in Fig 1, HbA1c levels in the OCCM group decreased significantly from baseline to 3 months and remained relatively stable at 6 months, whereas HbA1c levels in the usual-care group changed only slightly over time. Compared with baseline, the differences in HbA1c levels between the two groups gradually narrowed during follow-up, suggesting that the intervention may be associated with improvements in participants’ HbA1c levels.

**Fig 1:**
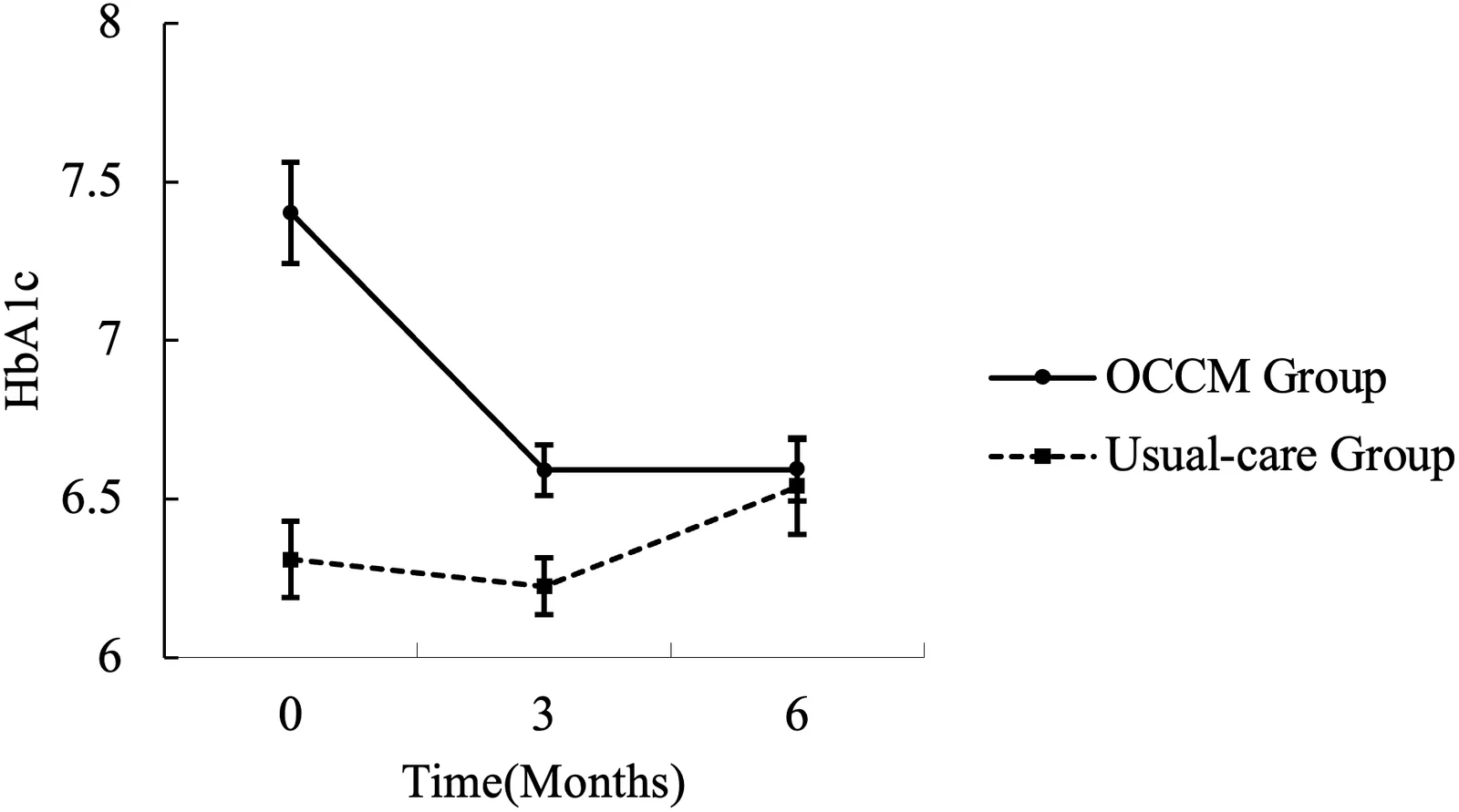
Changes in HbA1c levels over time in the OCCM and Usual-care groups (Mean ± SE).

The curves show the changes in mean HbA1c levels in the OCCM and usual-care groups from baseline to 3 and 6 months. Error bars represent SE. OCCM, Online Co-care Management; SE, standard error.

The repeated measures ANOVA results are presented in Table 3. Mauchly’s test indicated a violation of sphericity; therefore, results corrected using Roy’s largest root were adopted.A significant time effect was observed for HbA1c (*F* = 10.608, *P* < 0.001), indicating that blood glucose levels changed over time in both groups. The group effect was also significant (*F* = 16.748, *P* < 0.001), suggesting overall differences in HbA1c levels between the OCCM and control groups after adjusting for time. In addition, a significant time × group interaction was detected (*F* = 14.546, *P* < 0.001), indicating that the pattern of HbA1c change over time differed according to the type of care received.

**Table 3:** Repeated-Measures ANOVA Results for the Two Groups.

Variable	Time effect		Group effect		Time × group interaction	
	*F*	*P*	*F*	*P*	*F*	*P*
HbA1c	10.608	<0.001	16.748	<0.001	14.546	<0.001
SBP	10.338	<0.001	12.778	<0.001	10.239	<0.001
DBP	2.666	0.074	3.946	0.048	7.718	<0.001
BMI	9.116	<0.001	0.286	0.594	21.932	<0.001
LDLC	0.233	0.684	15.961	<0.001	0.207	0.813
HDLC	0.801	0.449	1.407	0.246	1.105	0.294
TC	8.489	0.001	27.311	<0.001	0.602	0.5
TG	5.078	0.007	0.914	0.34	0.664	0.514
eGFR	3.034	0.051	0.487	0.486	0.821	0.437
UA	2.483	0.085	7.251	0.008	0.565	0.568

Abbreviations used: HbA1c, hemoglobin A1c; BMI, Body Mass Index; SBP, Systolic Blood Pressure; DBP, Diastolic Blood Pressure; LDLC, Low-Density Lipoprotein Cholesterol; HDLC, High-Density Lipoprotein Cholesterol; TC, Total Cholesterol; TG, Triglycerides; eGFR, estimated glomerular filtration rate; ALT, alanine aminotransferase;AST, aspartate aminotransferase. UA, uric acid.

### Secondary outcome

As presented in Table 3, significant time effects were observed for systolic blood pressure (*F* = 10.338, *P* < 0.001), BMI (*F* = 9.116, *P* < 0.001), total cholesterol (TC, *F* = 8.489, *P* = 0.001), and triglycerides (TG, *F* = 5.078, *P* = 0.007), indicating that these metabolic indicators changed over the follow-up period in both groups. Significant group effects were observed for systolic blood pressure (*F* = 12.778, *P* < 0.001), LDL-C (*F* = 15.961, *P* < 0.001), total cholesterol (*F* = 27.311, *P* < 0.001), and uric acid (UA, *F* = 7.251, *P* = 0.008), reflecting overall differences between the OCCM and control groups independent of time. Significant time × group interactions were found for diastolic blood pressure (DBP, *F* = 7.718, *P* < 0.001) and BMI (*F* = 21.932, *P* < 0.001), suggesting that the trajectories of these indicators over time differed according to the care model. No significant time, group, or interaction effects were observed for eGFR (*P* > 0.05), indicating kidney function remained stable during follow-up. Descriptive statistics for secondary outcomes are shown in Table 2.

## Discussion

This study evaluated the short-term association between the Online Co-care Management (OCCM) model and changes in glycemic and metabolic indicators among patients with type 2 diabetes mellitus. Using a retrospective cohort with a 1:1 propensity score-matched usual-care control group, we analyzed follow-up data at baseline, 3 months, and 6 months.Compared with usual outpatient follow-up, the OCCM was associated with more favorable changes in HbA1c, BMI, blood pressure, and lipid profiles during the 6-month follow-up period. No significant timeeffect, groupeffect, or time × group interaction effect was observed for eGFR. These findings suggest that structured collaborative digital care may serve as a practical approach to supporting short-term glycemic and metabolic management in real-world clinical settings.

The primary clinical objective of diabetes management is the stabilization of blood glucose to prevent complications. HbA1c remains the “gold standard” for assessing the adequacy of glycemic control [15]. In this study, HbA1c in the OCCM group decreased from 7.403 ± 1.748% at baseline to 6.591 ± 0.871% at 3 months and 6.593 ± 1.056% at 6 months, while the control group showed smaller changes (6.310 ± 1.277% to 6.225 ± 1.017% and 6.534 ± 1.598%, respectively. achieving the optimal target of <7% recommended by diabetes guidelines [16].These results suggest that OCCM is correlated with a larger short-term decrease in HbA1c than usual care within a 6-month follow-up period. Notably, every 1% reduction in mean HbA1c is associated with a 21% reduction in the risk of diabetes-related complications [17]. Previous studies havealso indicated that mobile-based interventions yield statistically significant improvements in glycemic control in the short term [18,19], and may affect fasting blood glucose through remote care and health education [20].Our findings further support the potential value of combining digital follow-up with professional collaborative care in routine diabetes management.

From a care-delivery perspective, OCCM provides continuous education, online communication, follow-up reminders, medication guidance, and individualized lifestyle support, which may improve treatment adherence and self-management. This pattern may be partly explained by the “one-stop service framework” involving physicians and diabetes specialist nurses. Real-time feedback from healthcare professionals may contribute to improved glycemic control, enhanced quality of life, and better self-management [21].From a biological perspective, insulin resistance and progressive β-cell dysfunction are central to the pathophysiology of T2DM, while chronic metabolic stress may further contribute to β-cell injury and disease progression [22]. Recent evidence suggests that pancreatic lysosomal stress may participate in diabetes pathogenesis by affecting cellular homeostasis, metabolic sensing, inflammatory responses, and β-cell function [23]. Although these mechanisms were not directly examined in this study, improved glycemic and metabolic control under OCCM may help reduce chronic metabolic burden, providing a possible biological context for interpreting the observed clinical changes.

A pivotal finding of this study is the significant improvement in non-glycemic metabolic risk factors, including blood pressure and lipid profiles. Although glycemic control remains the core of traditional diabetes management, cardiovascular disease has become the leading cause of morbidity and mortality in T2DM patients. Clinical practice emphasizes gradual weight reduction and metabolic risk control, with BMI serving as a simple and practical indicator for routine follow-up [24–26]. More favorable changes in SBP, DBP, BMI, TC, LDL-C, TG, and UA were observed in the OCCM group than usual-care group. Time effects were also significant for SBP, BMI, TC, and TG, indicating changes over the follow-up period across both groups. Changes in the usual-care group were relatively small, suggesting that the structured, multidisciplinary OCCM approach may facilitate more consistent improvement in metabolic risk factors.Effective weight control aids in blood glucose management, with a 3% to 5% reduction in weight yielding significant clinical benefits [6,27]. These findings suggest that OCCM may help extend diabetes care beyond a glucocentric approach toward broader cardiometabolic risk management.

From the perspective of comprehensive cardiometabolic risk management, T2DM care should also consider common metabolic comorbidities, particularly metabolic dysfunction-associated steatotic liver disease (MASLD). T2DM and obesity are important cardiometabolic risk factors for the development and progression of MASLD [28]. Recent guidelines and reviews emphasize that patients with MASLD, especially those with metabolic risk factors, require screening, risk stratification, lifestyle modification, cardiometabolic risk control, and individualized multidisciplinary follow-up [28,29]. Although MASLD and liver-related outcomes were not directly assessed in the present study, the observed changes in HbA1c, BMI, blood pressure, and lipid profiles indicate that OCCM is aligned with the broader goal of integrated metabolic risk management in patients with T2DM. Future studies should include liver-related indicators, hepatic steatosis, and fibrosis assessment to further evaluate the potential role of OCCM in the co-management of T2DM and MASLD.

Lifestyle intervention remains central to both T2DM and MASLD management. Compared with conventional outpatient advice, structured digital lifestyle interventions usually provide more continuous and structured support by combining disease education, dietary and physical activity goals, weight monitoring, behavioral support, and regular feedback. Recent studies have shown that digital lifestyle or eHealth interventions can support weight loss and improve metabolic outcomes through education, self-regulation tools, health coaching, and remote follow-up [22,30]. The OCCM model shares these elements but further integrates physician-led treatment adjustment, diabetes specialist nurse education, electronic health records, and online multidisciplinary communication. This integrated structure may partly explain why OCCM was associated with short-term improvements in both glycemic and non-glycemic metabolic indicators.

Despite the increased intensity of management and significant improvements in metabolic indices, no statistically significant changes were observed in kidney function (eGFR, UA) over the six-month follow-up period. Given that many patients with T2DM are older and may be more vulnerable to medication-related risks or hypoglycemia, continued monitoring remains necessary during collaborative diabetes management. The professional follow-up and remote communication provided in the OCCM model may help support timely identification of abnormal clinical changes, but further studies with longer follow-up and detailed adverse event data are needed to evaluate safety more fully.

### Limitations

Several limitations should be acknowledged. First, although propensity score matching was used to reduce baseline imbalance between groups, unmeasured confounding may still exist. Second, the study was based on routinely collected clinical follow-up records, and selection bias cannot be fully excluded. Patients who participated in OCCM may have had greater willingness or ability to use digital communication tools and attend regular follow-up visits. Third, the follow-up period was limited to 6 months, which restricts the assessment of long-term sustainability. Fourth, this study did not include detailed adverse event data, such as hypoglycemia, hospitalization, emergency visits, or treatment discontinuation. Therefore, safety should be interpreted with caution. Future multicenter prospective studies with larger sample sizes, longer follow-up, and assessments of adverse events, liver-related outcomes, and mechanistic biomarkersare needed to further evaluate the clinical value and cost-effectiveness of OCCM.

## Conclusion

Compared with usual outpatient follow-up, the OCCM model was associated with more favorable short-term changes in HbA1c, BMI, blood pressure, and lipid profiles among patients with T2DM. These findings support the potential value of structured collaborative digital care in routine diabetes management. However, because this was a retrospective observational study, the results should be interpreted as associations rather than causal effects. Further prospective studies with longer follow-up are needed to confirm these findings and to evaluate long-term effectiveness and safety.

## Supporting information

S1 File Supplementary information on participant selection process, pre-matching characteristics and outcomes, and missing data handling. (DOCX)
