# Supplementary material for: Online co-care management and glycemic control in patients with type 2 diabetes mellitus: A propensity score-matched retrospective cohort study
Source: PLoS One. 2026 Nov 2;21(11):e0360677. doi: 10.1371/journal.pone.0360677 (PMC13726964; doi:10.1371/journal.pone.0360677)
Supplement: Supplementary file 2 [file pone.0360677.s003.docx]

**Manuscript Number: PONE-D-26-04021**

**Journal: PLOS ONE**

**Dear Editor and Reviewers:**

We sincerely thank the Editor and Reviewers for the careful evaluation of our manuscript and for the constructive comments. We greatly appreciate the opportunity to revise our work. Your suggestions helped us recognize important limitations in the original version, particularly regarding study design, cohort description, time-point definition, statistical analyses, and result reporting.

In response, we have supplemented the usual-care control group, clarified the study timeline and follow-up, implemented 1:1 propensity score matching, and updated statistical analyses including repeated-measures ANOVA. All changes have been highlighted in the revised manuscript.

**Academic Editor**

Dear Dr. Lanlan Chen,

Thank you for your careful review and for providing constructive comments. We have revised our manuscript thoroughly in response to all points raised. Please see our point-by-point responses below:

**1. Please ensure that your manuscript meets PLOS ONE's style requirements, including those for file naming. The PLOS ONE style templates can be found at**

[**https://journals.plos.org/plosone/s/file?id=wjVg/PLOSOne_formatting_sample_main_body.pdf**](https://journals.plos.org/plosone/s/file?id=wjVg/PLOSOne_formatting_sample_main_body.pdf) **and** [**https://journals.plos.org/plosone/s/file?id=ba62/PLOSOne_formatting_sample_title_authors_affiliations.pdf**](https://journals.plos.org/plosone/s/file?id=ba62/PLOSOne_formatting_sample_title_authors_affiliations.pdf) **Response:**

Thank you very much for your kind reminder We carefully checked our manuscript to ensure it meets PLOS ONE style and formatting requirements.

**2. One of the noted authors is a group or consortium. In addition to naming the author group, please list the individual authors and affiliations within this group in the acknowledgments section of your manuscript. Please also indicate clearly a lead author for this group along with a contact email address.**

**Response:**

Thank you very much for your careful attention to this matter. We acknowledge that there was no group author for this manuscript, and this oversight will be corrected in the system when the revised manuscript is uploaded. All individual authors and affiliations are correctly listed, and the corresponding author is clearly indicated. We appreciate your careful review and guidance on this matter.

**3. We note that you have indicated that there are restrictions to data sharing for this study. For studies involving human research participant data or other sensitive data, we encourage authors to share de-identified or anonymized data. However, when data cannot be publicly shared for ethical reasons, we allow authors to make their data sets available upon request. For information on unacceptable data access restrictions, please see** [**http://journals.plos.org/plosone/s/data-availability#loc-unacceptable-data-access-restrictions**](http://journals.plos.org/plosone/s/data-availability)**.**

**Response:**

Thank you for this suggestion. We have clarified the ethical and legal restrictions regarding data sharing. The clinical follow-up dataset contains potentially identifiable patient information; thus, it cannot be publicly shared due to patient privacy protections approved by the Institutional Review Board of Beijing Hospital of Traditional Chinese and Western Medicine. Researchers may request de-identified data by contacting the ethics committee :Address: No. 3 Yongding Road East Street, Haidian District, Beijing Email: zxyxck@163.com 24-hour hotline: +86 13521828421

**4. Please include captions for your Supporting Information files at the end of your manuscript, and update any in-text citations to match accordingly. Please see our Supporting Information guidelines for more information:** [**http://journals.plos.org/plosone/s/supporting-information**](http://journals.plos.org/plosone/s/supporting-information)**.**

**Response:**

Thank you for your kind reminder. We have reorganized and integrated the statistical results in the revised manuscript to provide a clearer and more informative presentation. The main descriptive and repeated-measures ANOVA results are now fully reported in Tables 2 and 3, and the figure captions have been updated for clarity. Following this integration, there is no longer a need for a supplementary table, and all key data are now presented within the main manuscript.

**5. If the reviewer comments include a recommendation to cite specific previously published works, please review and evaluate these publications to determine whether they are relevant and should be cited. There is no requirement to cite these works unless the editor has indicated otherwise.**

**Response:**

Thank you for your kind reminder. We have carefully considered your suggestion and agree that some of the references previously used are outdated for the current clinical context. We believe it is necessary to update the references with more contemporary guidelines on diabetes diagnosis and management to provide an accurate and current clinical framing. We reviewed the suggested references and incorporated contemporary diabetes diagnosis and management guidelines (2020 China Guideline for the Prevention and Treatment of T2DM, published in 2021)

**Reviewer #1: Reviewer Comment**

***Major comments***

**1. The study timeline is internally inconsistent and must be clarified before the results can be interpreted.**

**The manuscript states that patients were retrospectively included from September 2022 to September 2025, but it also states that the recruitment period was from 08/03/2024 to 08/06/2024. At the same time, the intervention description says HbA1c, blood glucose, and liver/kidney function were assessed annually over a three-year period. These three statements do not fit together logically as currently written. The authors must clearly distinguish: (1) the period of record screening, (2) the actual cohort entry/baseline date, (3) the definition of T1/T2/T3, and (4) the duration of follow-up for each participant. Without this clarification, the study design is not interpretable.**

**Response:**

Thank you for pointing this out. We have revised the Methods and Results sections to clarify the study timeline and the definition of follow-up time points.

(1) the period of record screening: We now state that all follow-up records of patients with type 2 diabetes from September 2022 to September 2025 were extracted. After applying the exclusion criteria and data cleaning procedures, eligible patients were included in the cohort.

(2) the actual cohort entry/baseline date: we clarified the follow-up time points throughout the manuscript. The three time points are now consistently defined as baseline, 3-month follow-up, and 6-month follow-up. These time points are labeled as T0, T1, and T2, respectively. We removed inconsistent descriptions related to annual follow-up or three-year outcome assessment.

(3) the definition of T1/T2/T3: eligible patients were included when they first met the inclusion criteria. This date was defined as the baseline (T0). Follow-up assessments were conducted at 3 months (T1) and 6 months (T2) after baseline.

(4) duration of follow-up: Each patient was followed from baseline to the earliest of the following: completion of 6-month follow-up, loss to follow-up, or study cutoff date. We removed inconsistent statements about annual assessment or three-year follow-up. (*Methods, page 2-3, lines 66-71，79–84; Results, page 4, lines 139–144).*

**2. The manuscript is essentially a single-arm longitudinal observational study, but the title and conclusions use overly causal language.**

**All analyses appear to be within one OCCM-managed cohort using repeated-measures ANOVA across timepoints, with no usual-care comparator, no external control. Under this design, the data support an association between follow-up under OCCM and improved metabolic indicators, but they do not establish the “effect” of OCCM in a strong causal sense. The title, objective, and conclusion should be reframed accordingly unless a comparator group exists and is fully reported.**

**Response:**

Thank you for this important comment. We fully agree that the original manuscript used overly causal language and that the absence of a comparator limited the interpretation of the findings. To address this concern, we made substantial revisions to the study design. Specifically, we added a usual-care control group consisting of patients with type 2 diabetes who received routine outpatient follow-up care during the same study period. We then used 1:1 propensity score matching to reduce baseline imbalance between the OCCM and usual-care groups. After matching, 240 patients were included in the final analysis, with 120 patients in each group. *（Methods, page 3, lines 79–84； page 4, lines 128–131; Results, page 4, lines 139–154.）*

We also revised the manuscript title and related wording to better reflect the revised study design and the observational nature of the data. The title was changed to “Online Co-care Management and Glycemic Control in Patients with Type 2 Diabetes Mellitus: A Propensity Score-Matched Retrospective Cohort Study.” In the abstract and introduction, we revised the objective to focus on whether OCCM was associated with changes in HbA1c and secondary metabolic indicators, rather than claiming a direct causal effect. *(Title, page 1, lines 1–2.)*

In addition, we revised the Results, Discussion, and Conclusion sections to avoid strong causal claims. We now describe the findings using association-based language, such as “was associated with” and “may provide practical support,” and focus on differences in longitudinal changes between the OCCM group and the matched usual-care group. These revisions ensure that the manuscript presents the findings in a way that is consistent with a propensity score-matched retrospective cohort study.

**3. Absence of a control group prevents clear attribution of the observed benefits**

**Related to my second concern, the absence of a control group makes it difficult to determine whether the observed improvements were attributable to the OCCM care model itself or simply to the effects of glucose-lowering therapy. Standard antidiabetic treatment alone may also lead to similar improvements in metabolic outcomes. Moreover, patients who were able to complete regular follow-up over a three-year period were likely to have better medication adherence than the general diabetic population, which may have further contributed to the favorable results. Given the large number of patients with diabetes, I believe that inclusion of an appropriate control group would be necessary to better substantiate the study findings. Importantly, this issue was not acknowledged by the authors in the limitations section.**

**Response:**

Thank you for this important and constructive comment. We agree that the absence of a control group in the original manuscript limited the interpretation of the observed improvements. To address this concern, we made substantial additional efforts to retrieve, screen, and clean clinical follow-up records from patients with type 2 diabetes who received usual outpatient follow-up care during the same study period. We then added these patients as a usual-care control group and revised the study design as a propensity score-matched retrospective cohort study. (*Methods, page 2, lines 66–71).*

Specifically, We screened 605 patients from 977 medical records according to the inclusion and exclusion criteria for the initial cohort, including 406 patients in the OCCM group and 199 patients in the usual-care group. We then performed 1:1 propensity score matching to reduce baseline differences between the two groups. After matching, 240 patients were included in the final analysis, with 120 patients in the OCCM group and 120 patients in the usual-care group. The propensity score model included age, sex, diabetes duration, smoking, drinking, medication use, and follow-up duration. (*Statistical analysis, page 4, lines 128–136; Results, page 4, lines 139–154.)*

**4. Cohort assembly and follow-up completeness are insufficiently reported, creating serious risk of selection bias.**

**The inclusion criteria require that patients “voluntarily complete all follow-ups,” which strongly suggests a completer-only analysis. If that is the case, the study may be enriched for adherent patients and may overestimate benefit. The manuscript should provide a patient flow diagram: number screened, excluded, lost to follow-up, and included in each timepoint analysis. It should also specify whether all 406 patients had complete data at all three timepoints.**

**Response:**

Thank you for this helpful and important suggestion. We agree that the previous wording “voluntarily complete all follow-ups” could be interpreted as a completer-only analysis and may raise concerns about selection bias. Therefore, we revised the inclusion criteria and removed this wording. In the revised manuscript, the inclusion criteria now focus on diagnosis of T2DM, diabetes duration ≥6 months, and availability of follow-up data during the study period. （*Methods, page 2, lines 69–71）.*

We also added a participant flowchart to clearly describe cohort assembly and patient selection. In the revised Results section, we report that 977 follow-up records of patients with type 2 diabetes from 2022 to 2025 were extracted. After excluding patients with dropout, loss to follow-up, extreme values, or records that met the exclusion criteria, 605 patients were included in the initial cohort, including 406 patients in the OCCM group and 199 patients in the usual-care group. After 1:1 propensity score matching, 240 patients remained in the final analysis, with 120 patients in each group. (*Supplement Figure 1)*

To further improve transparency, we clarified that the repeated-measures analysis was conducted using the matched cohort with available follow-up data at baseline, 3 months, and 6 months. We also defined the three time points consistently as baseline, 3-month follow-up, and 6-month follow-up. This revision was intended to make the cohort construction and follow-up structure clearer and to avoid ambiguity regarding the population included in the final analysis.

**5. The safety conclusion is overstated.**

**The manuscript concludes that OCCM is “safe” because ALT, AST, eGFR, and serum creatinine did not change significantly. However, absence of statistically significant change in a limited set of annual laboratory values is not equivalent to demonstrating safety. No adverse event data are reported, and there is no information on hypoglycemia, hospitalization, emergency visits, treatment discontinuation, or other clinically relevant harms. The authors should soften the wording to state that no obvious deterioration was observed in the measured liver and kidney laboratory indices during follow-up.**

**Response:**

Thank you for this important and careful comment. We agree that the absence of statistically significant changes in a limited set of laboratory indicators should not be interpreted as evidence that the OCCM model is safe. In the revised manuscript, we have removed the strong safety-related conclusion and no longer state that OCCM is “safe” based only on laboratory findings. （*Discussion, page 6, lines220-224, 230–236）*

We also revised the Results and Discussion sections to use more cautious wording. In the Results section, we now only state that no significant time, group, or time × group interaction effects were observed for eGFR during the 6-month follow-up. In the Discussion section, we further clarify that stable eGFR suggests relatively stable renal function in this cohort, but it should not be interpreted as definitive evidence of safety. （*Results, page 5, lines 164–172; Discussion, page 6, lines 230–236.）*

In addition, we have acknowledged the lack of adverse event data as an important limitation. Specifically, we now state that adverse events such as hypoglycemia, hospitalization, emergency visits, treatment discontinuation, and medication-related harms were not assessed in this study. Therefore, safety should be interpreted with caution, and future studies with longer follow-up and detailed adverse event data are needed to evaluate safety more fully. *（Limitations, page 6-7, lines 238–247.）*

**6. There are apparent data-quality and reporting errors that need careful verification.**

**At least one numeric entry appears implausible: baseline LDL-C is reported as 2.577 ± 6.069, whereas subsequent SDs are 0.866 and 0.904; this strongly suggests a data-entry, unit, or formatting error. Table 1 also appears to contain labeling/percentage problems and unclear medication abbreviations (for example, “Sus”), and the Results text mentions “Alcohol use duration” and “Smoking duration,” whereas the table presents only yes/no categories. These issues reduce confidence in the dataset and require a full data audit.**

**Response:**

Thank you for identifying these important issues. We fully agree that these data-quality and reporting problems required careful verification. In response, we conducted a full review of the dataset, tables, variable labels, abbreviations, and the corresponding descriptions in the Results section. We corrected formatting and reporting errors and revised the tables to ensure consistency between the data, table labels, and text*..*

Regarding the reviewer’s concern about the implausible LDL-C value, we rechecked the original data and revised the outcome table according to the updated dataset and analysis. In the revised Table 2, LDL-C values are now reported consistently across the three time points for both groups. The revised results are presented as mean ± standard deviation, and the corresponding repeated-measures ANOVA results are reported in Table 3. *（Table 2, pages11, lines 377; Table 3, pages 12, lines 382.）*

We also revised Table 1 to present the matched cohort more clearly. The revised Table 1 now includes 240 patients after propensity score matching, with 120 patients in the OCCM group and 120 patients in the usual-care group. Baseline characteristics, including age, diabetes duration, sex, drinking, smoking, hypertension, hyperlipidemia, and antidiabetic medication use, are now reported using consistent labels and percentages. *（Table 1, pages 10, lines 375）*

In addition, we corrected unclear medication abbreviations. Specifically, “Sus” was revised to “Sulfonylureas,” and “DPP4i” was revised to “DPP-4 inhibitors.” *（Table 1, pages 11, lines 375*

We also corrected the inconsistency between the Results text and the table regarding smoking and drinking variables. The original wording could be interpreted as duration of smoking or drinking. In the revised manuscript, these variables are consistently described as smoking (yes/no) and drinking (yes/no). （*Methods, page 4, lines 117–118）*

**7. The reporting is incomplete in several places.**

**The manuscript states that results of body-measurement ANOVA are presented in Supplement Table 1, but the main narrative does not provide sufficient numeric detail. The Methods also state that satisfaction scores were collected, yet satisfaction results are not reported in the Results section. If these variables were collected, they should either be reported or removed from the Methods.**

**Response:**

Thank you for this helpful comment. We agree that the original manuscript did not provide sufficient numerical details in the main Results section. In the revised manuscript, we reorganized the Results section and provided a clearer description of the primary and secondary outcomes. The primary outcome is now clearly defined as glycated hemoglobin (HbA1c), and the secondary outcomes include body mass index (BMI), blood pressure, lipid profiles, and renal function indicators. *（Methods, page 3, lines 83-86）*

We added descriptive results for HbA1c at baseline, 3 months, and 6 months in both the OCCM and usual-care groups. Specifically, HbA1c in the OCCM group decreased from 7.403 ± 1.748% at baseline to 6.591 ± 0.871% at 3 months and 6.593 ± 1.056% at 6 months. In the usual-care group, HbA1c changed from 6.310 ± 1.277% at baseline to 6.225 ± 1.017% at 3 months and 6.534 ± 1.598% at 6 months. These values are now reported in the main text and Table 2. *（Results, page 5, lines 157–160; Table 2, pages 11, lines 377.）*

We also reported repeated-measures ANOVA results in the main Results section. For HbA1c, the time effect, group effect, and time × group interaction were all significant. For secondary outcomes, we reported significant time effects for systolic blood pressure, BMI, total cholesterol, and triglycerides; significant group effects for systolic blood pressure, LDL-C, total cholesterol, and uric acid; and significant time × group interactions for diastolic blood pressure and BMI. These results are now presented in the main text and Table 3.

Regarding satisfaction scores, we carefully reviewed the variables included in the revised analysis. Because satisfaction scores were not included in the final analysis, we removed the corresponding description from the Methods section to avoid inconsistency between the Methods and Results. The revised Methods section now only includes variables that are reported in the Results and tables.

In addition, we removed the previous reference to Supplement Table 1 and revised the presentation of outcome data. The main outcome data are now shown in Table 2, and the repeated-measures ANOVA results are shown in Table 3. This revision provides more complete numerical details in the main manuscript and improves consistency between the Methods, Results, and tables.

These revisions provide a clearer and more complete report of the primary and secondary outcomes, remove variables that were not included in the final analysis, and improve the consistency of the manuscript.

***Minor comments***

**1. Language editing is needed throughout.**

**There are multiple grammatical and typographical problems, such as duplicated wording (“for type 2 diabetes for type 2 diabetes”), “repeatedmeasures,” “showed a improve trend,” inconsistent capitalization, and inconsistent spacing/punctuation. A thorough professional English edit is necessary**.

**Response:**

Thank you for this suggestion. We have carefully revised the manuscript throughout for grammar, spelling, and typographical errors. Specifically, we:

(1) Corrected duplicated phrases (e.g., “for type 2 diabetes for type 2 diabetes”).

(2) Fixed spacing and punctuation issues, ensuring consistent formatting.

(3) Corrected verb usage and tense, including “showed an improved trend.”

(4) Standardized capitalization throughout headings, tables, and the main text.

(5) Ensured consistent terminology for key variables and statistical terms, including “repeated-measures ANOVA.”

**2. The definition of timepoints should be made explicit.**

**The abstract refers to “baseline to year 3,” while the Methods describe analyses at year 1, year 2, and year 3, and the tables use T1/T2/T3. It is unclear whether T1 is baseline or year 1. This should be standardized throughout the manuscript.**

**Response:**

Thank you for this comment. We have standardized the timepoint definitions throughout the manuscript. Baseline is now consistently defined as T0, with follow-up assessments at 3 months (T1) and 6 months (T2). The abstract, Methods, Results, tables, and figures have been updated to reflect this consistent labeling. This ensures that readers can clearly interpret the timing of measurements and longitudinal changes.

**3. Outcome definitions should be standardized.**

**The manuscript uses “fasting blood glucose” in the abstract but “preprandial blood glucose” in the Methods and Results. The authors should define exactly what was measured and at what time relative to meals.**

**Response:**

Thank you for this comment. We have standardized the outcome definitions throughout the manuscript. The primary glycemic outcome is now consistently reported as glycated hemoglobin (HbA1c), which reflects average blood glucose over the previous 2–3 months. Fasting blood glucose was no longer reported as a primary or secondary outcome to avoid inconsistency. All references in the abstract, Methods, Results, tables, and figures now use HbA1c consistently.

**4. Figures are not sufficiently informative.**

**The current bar plots are visually simple but do not show individual variability over time, exact pairwise significance, or sample size at each timepoint. Line plots or spaghetti/mean trend plots with confidence intervals may be more informative for longitudinal data. The supplementary table referenced in the text should also be provided clearly.**

**Response:**

Thank you for this helpful suggestion. We agree that the original figures were not sufficiently informative for presenting longitudinal data. In the revised manuscript, we improved the visual presentation of the study results in two ways. First, we added Supplement Figure 1, a flowchart of study population selection, to clearly show the numbers of records screened, excluded, included in the initial cohort, and retained after propensity score matching. This revision improves the transparency of cohort assembly. （*Supplement Figure 1.）*

Second, we replaced the previous simple presentation of glycemic change with Figure 2, which shows the mean trend of HbA1c over time in the OCCM and usual-care groups. This figure more clearly illustrates the longitudinal pattern of change from baseline to 3 months and 6 months in the two groups. We also described the main trend shown in the figure in the Results section to improve consistency between the text and the visual presentation. （*Figure 2.）*

In addition, we revised the outcome tables to provide the detailed numerical results that were not fully captured by the original figures. Table 2 now reports the descriptive values of HbA1c and secondary outcomes at each time point, and Table 3 reports the repeated-measures ANOVA results, including time effects, group effects, and time × group interactions. We believe that the combination of revised figures and detailed tables provides a clearer and more informative presentation of the longitudinal data. （*Table 2, pages 11, lines 377; Table 3, pages 12, lines 382.）*

**5. Some references are outdated for current clinical framing.**

**For example, the diagnostic criterion citation is based on a 1998 WHO report. The authors should consider citing more contemporary diabetes diagnostic and management guidelines alongside historical references.**

**Response:**

Thank you for this suggestion. In the revised manuscript, we updated the diagnostic standard to follow the “Guideline for the prevention and treatment of type 2 diabetes mellitus in China (2020 edition)” published by the Chinese Journal of Diabetes in 2021. （*Methods, page 2, lines 69-70）*

**We sincerely appreciate the time and effort that the Editor and Reviewers have devoted to evaluating our manuscript. We greatly value this opportunity, and we made every effort to supplement the control group, refine the study design, implement propensity score matching, and update statistical analyses. The reviewers’ thoughtful suggestions helped us substantially improve the clarity, rigor, and transparency of our work. We hope that the revised manuscript and our responses meet with your approval. Once again, we sincerely thank the Editor and Reviewers for their constructive comments and guidance.**
