# Supplementary material for: Online co-care management and glycemic control in patients with type 2 diabetes mellitus: A propensity score-matched retrospective cohort study
Source: PLoS One. 2026 Nov 2;21(11):e0360677. doi: 10.1371/journal.pone.0360677 (PMC13726964; doi:10.1371/journal.pone.0360677)
Supplement: Supplementary file 3 [file pone.0360677.s004.docx]

**Manuscript Number: PONE-D-26-04021**

**Journal: PLOS ONE**

**Dear Editor and Reviewers:**

Thank you very much for your careful evaluation of our revised manuscript and for your constructive suggestions. We are pleased to know that most of the concerns have been addressed. We have further revised the Discussion section according to your comments and also carefully reviewed the reference list. All changes have been highlighted in the revised manuscript.

**Dear Maidelyn R. Peregrin:**

**1. Please ensure that you refer to Figure 1 in your text as, if accepted, production will need this reference to link the reader to the figure.**

**Response:**

Thank you for this comment. We have revised the figure arrangement in the manuscript. The original Fig 1, which presented a schematic overview of the OCCM model, has been removed because the OCCM model is already sufficiently described in the main text. Accordingly, the former Fig 2, showing the changes in HbA1c levels over time, has been renumbered as Fig 1, and its corresponding in-text citation has been updated accordingly. *(,Results, page5, lines 164)*

In addition, the former Supplementary Figure 1 has been incorporated into S1 File, together with the baseline characteristics before propensity score matching, the primary and secondary outcome measures before matching, and the description of missing data handling. The corresponding reference in the main text has also been updated to S1 File. （*Results, page4, lines 147;* *Supporting information, page8, lines 285-826）*

**Academic Editor：**

**1. The discussion is weak, and the authors should discuss the co-management of T2D and MASLD (PMID: 40510733; PMID: 41668679).**

**Response:**

Thank you for this important suggestion. We agree that the Discussion section should better reflect the broader context of cardiometabolic risk management in patients with T2DM. We have added a new discussion on the co-management of T2DM and MASLD. In the revised manuscript, we discuss that T2DM and obesity are major metabolic risk factors for the development and progression of MASLD, and that patients with MASLD, especially those with metabolic risk factors, require screening, risk stratification, lifestyle modification, cardiometabolic risk control, and individualized multidisciplinary follow-up. We have also clarified that MASLD and liver-related outcomes were not directly assessed in the present study; therefore, related interpretations should be considered contextual rather than direct evidence from this study. The recommended references have been added to support this discussion [28, 29]. （*Discusion, page6-7, lines 237-247）*

**2. Meanwhile, please discuss some potential mechanisms, e.g. how pancreatic lysosomal stress affects diabetes (PMID: 39512752).**

**Response:**

Thank you for your helpful comment. This was indeed an aspect that needed to be strengthened in our manuscript. In the Discussion section, we have expanded the explanation from both a “care-delivery perspective” and a “biological perspective,” added content on potential mechanisms. We have emphasized that these mechanisms were not directly examined in the present study and should be interpreted as a possible biological context for the observed clinical changes. The recommended reference has been added [23]. (*Discusion, page6, lines 211-223）*

**3. Some lifestyle intervention strategies should be compared (PMID: 40588354; PMID: 40204429).**

**Response:**

Thank you for this constructive suggestion. We now discuss that, compared with conventional outpatient advice, structured digital lifestyle interventions usually provide more continuous and structured support by combining disease education, dietary and physical activity goals, weight monitoring, behavioral support, and regular feedback. We further discuss that the OCCM model shares these elements but additionally integrates physician-led treatment adjustment, diabetes specialist nurse education, electronic health records, and online multidisciplinary communication. This integrated structure may partly explain why OCCM was associated with short-term improvements in both glycemic and non-glycemic metabolic indicators. The recommended references have been added [22, 30]. (*Discusion, page7, lines 248-257）*

**4. Reference list check**

**Response:**

Thank you for the journal’s reminder regarding the reference list. We have carefully reviewed all references and did not identify any retracted articles. To improve the accuracy and relevance of the reference list, we replaced the previous reference [9] with the updated ADA Standards of Care in Diabetes—2026. This new reference more directly supports our discussion of coordinated interprofessional diabetes care, including the involvement of clinicians, nurses, diabetes care and education specialists, dietitians, pharmacists, and other health professionals in diabetes management.

**We sincerely appreciate the editor’s valuable comments, which have helped us further improve the depth, clinical relevance, and overall quality of the manuscript. In addition, we made several minor revisions to improve wording, clarity, and grammatical accuracy throughout the manuscript. These changes do not affect the study design, results, or conclusions, and all revised text has been marked in red font.**
